# OA13.01. Mind-Body Medicine Skills training for self-care and emotional well-being in medical students

**DOI:** 10.1186/1472-6882-12-S1-O49

**Published:** 2012-06-12

**Authors:** M Dutton, A Prashar, G Romero, J Talley, H Amri, A Haramati, N Harazduk

**Affiliations:** 1 Georgetown University Medical Center, Washington, D.C., USA

## Purpose

The purpose of this study was to examine the effect of a Mind-Body Medicine program for enhancing self-care and well-being among first year medical students. Depression and anxiety are common among medical students (West et al, 2011) and physicians (Krasner et al, 2009). Further, distress among medical students is associated with poor patient care (Dyrbye et al, 2010; Krasner et al, 2009). Thus, prevention and self-care have become central to medical education. One approach to developing self-care competencies among medical students is through enhancing mind-body skills. At the GUMC, these skills are taught in a Mind-Body Skills (MBMS) program. The 11-week MBMS program includes teaching of self-awareness, relaxation, meditation, guided imagery, biofeedback, physical exercise, art, music and movement. The program promotes self-care within a context of group support.

## Methods

First-year medical students, 34 females and 25 males, from a class of 192 students elected the MBMS and were administered pre- and post-test assessment questionnaires measuring depression and anxiety (BSI; Derogatis, 1983), physical health symptoms (Wahler, 1983) and mindfulness (5FMS; Baer, 2006).

## Results

Bonferroni-corrected results show significant improvements in students' pre-post levels of depression (M=.78 vs.58, SD=.86 and .73, respectively; t=3.08, p<.003) and anxiety (M=40.95 vs. 33.17, SD=10.97 and SD=9.32, respectively; t=6.46, df=58, p<.001) with a trend for physical health (M=23.54 vs. 19.98, SD=14.00 and 15.37, respectively; t=2.20, p<.03). Mindfulness skills also increased (M=121.05 vs. 132.68, SD=16.48 and 18.31, respectively). Controlling for baseline levels, increases in mindfulness skills predicted post-test levels of depression and anxiety, accounting for an additional 10% and 12% of the variance.

## Conclusion

The GUMC MBMS course for medical students can lay a foundation for prevention of stress often experienced by medical professionals (Krasner, 2009). Specifically, MBMS was associated with reduction in distress, and change in mindfulness skills appear to account, in part, for improvements in depression and anxiety.

